# Phosphorus loss assessment tools: a review of underlying concepts and applicability in cold climates

**DOI:** 10.1007/s11356-019-06800-9

**Published:** 2019-12-09

**Authors:** Reza Habibiandehkordi, D. Keith Reid, Pradeep K. Goel, Asim Biswas

**Affiliations:** 1 Agriculture and Agri-Food Canada, Guelph, ON N1G 4S9 Canada; 2 Ministry of the Environment, Conservation and Parks, Etobicoke, ON M9P 3V6 Canada; 3 School of Environmental Sciences, University of Guelph, Guelph, ON N1G 2W1 Canada

**Keywords:** Cold climates, Critical source areas, Phosphorus loss assessment tools, Runoff, Water quality

## Abstract

Identifying critical source areas (CSAs) of a watershed by phosphorus (P) loss assessment tools is essential for optimal placement of beneficial management practices (BMPs) to address diffuse P pollution. However, lack of significant progress in tackling diffuse P pollution could be, in part, associated with inefficacy of P loss assessment tools for accurately identifying CSAs. Phosphorus loss assessment tools have been developed to simulate P loss from the landscape where runoff is mainly driven by rainfall events. Therefore, they may underperform in cold climates where the land is often frozen during winter and runoff is dominated by snowmelt. This paper (i) reviews the strengths and weaknesses of current P loss assessment tools and their underlying assumptions in simulating soil P dynamics and P transfer to runoff, and (ii) highlights a number of challenges associated with modeling P transfer from agricultural land to surface waters in cold climates. Current P loss assessment tools do not appear to fully represent hydrological and biogeochemical processes responsible for P loss from CSAs, particularly in cold climates. Effort should be made to develop P loss assessment tools that are capable of considering P dynamics through the landscape as a result of abiotic perturbations that are common in cold climates, predicting runoff and P movement over frozen/partially frozen soils, and considering material-P connectivity between landscape and surface waters. Evaluating P loss assessment tools with water quality data is necessary to ensure such modifications result in improved identification of CSAs.

## Introduction

Phosphorus (P) export from agricultural land has been identified as a primary cause of water quality impairment as P plays an important role in freshwater eutrophication (Schindler et al. 2012; Watson et al. 2016). Eutrophication poses several consequences to aquatic ecosystems, such as increased frequency of harmful planktonic and benthic algal blooms and hypoxia, reductions in water transparency and aquatic biodiversity, and increased costs of drinking water treatment (Sharpley et al. 2003a; Smith and Schindler 2009; Watson et al. 2016). Despite the development and implementation of a wide range of beneficial management practices (BMPs) to reduce P export, little or no significant improvement in water quality has been reported for some of the world’s largest freshwater resources including the Great Lakes of North America (Schindler et al. 2012; Sharpley et al. 2015; Watson et al. 2016). Lack of progress in reducing diffuse P pollution could be, in part, associated with inappropriate placement or selection of BMPs within agricultural watersheds, the lag time in water quality response to BMPs, climate variability, and inadequate P management regulations or landowners’ commitment (Sharpley et al. 2013).

Past research has shown disproportionate losses of P from certain areas of a watershed known as critical source areas (CSAs) where large P sources could coincide with hydrological flows across small portions of the watershed (Ghebremichael et al. 2010; Li et al. 2017). For example, Ghebremichael et al. (2010) found that 80% of the total P loss from the Rock River watershed in Vermont, USA is generated from only 24% of the watershed area. Identifying such CSAs or “hotspots” in a watershed by P loss assessment tools is essential for optimal placement of BMPs to address P pollution that can lead to eutrophication (Kleinman et al. 2011a).

Several approaches ranging from simple, easy-to-use P indices (e.g., the Pennsylvania P index (Weld et al. 2002); the Kentucky P index (Bolster 2011)) to complex process-based models (e.g., the soil and water assessment tool (SWAT) (Arnold et al. 1998); the annualized agricultural non-point source pollution (AnnAGNPS) (Bosch et al. 1998)) have been developed assuming runoff is generated primarily through rainfall-runoff events, as is the case in most warm temperate climates, but these tools have also been used in cold climates. The term “cold climates” used throughout this paper refers to the regions with prolonged periods of average daily temperatures below zero degrees Celsius during winter where a greater proportion of runoff is generated during snowmelt than rainfall events (e.g., Northern US states, Canada, Northern Europe). However, the lack of evidence for downstream water quality improvement where P loss assessment tools have been used to place BMPs leads us to consider whether or not these tools have failed to accurately delineate CSAs.

Failure to accurately identify CSAs could be associated with inability of P loss assessment tools to fully account for the hydrological and biogeochemical processes responsible for P loss, particularly in cold climates. Phosphorus loss assessment tools have been modified and extensively used worldwide to simulate P loss from the landscape (e.g., Beaulieu et al. 2006; Bechmann et al. 2007; Collick et al. 2016; Das et al. 2008; Easton et al. 2008; Li et al. 2015; Li et al. 2017; Niraula et al. 2013; Reid 2011), and efforts have been made to revise some of the empirically based equations and/or their underlying assumptions (e.g., Collick et al. 2016; Bolster and Vadas 2018; Mekonnen et al. 2017; Vadas and White 2010; Vadas et al. 2013). However, there is little information in the literature on the effectiveness of P loss assessment tools for simulating P loss during snowmelt runoff events; neither is there any thorough review regarding applicability of the underlying concepts of P loss assessment tools in cold climates. This manuscript (i) reviews the strengths and weaknesses of current P loss assessment tools and their underlying concepts in delineating CSAs of agricultural watersheds, and (ii) highlights a number of challenges associated with modeling P transfer from agricultural land to surface waters in cold climates. The manuscript was structured to describe how soil P dynamics and runoff contributing areas are simulated by P loss assessment tools in agricultural watersheds.

## Simulating soil P dynamics in agricultural watersheds by P loss assessment tools

### Process-based P loss assessment tools

Commonly used process–based tools, such as SWAT and AnnAGNPS, use the conceptual P model of Jones et al. (1984), which was originally developed for the erosion productivity impact calculator (EPIC) model (Williams et al. 1984) to simulate the processes responsible for soil P dynamics. Jones et al. (1984) conceptualized P cycling in soils by dividing soil total P into the inorganic (labile, active, and stable) and organic (fresh and stable/humic) P pools. However, these P routines and their underlying assumptions may not be applicable beyond a narrow range of soils, climates, and nutrient management practices (Vadas and White 2010; Vadas et al. 2013; Bolster and Vadas 2018).

For example, according to Jones et al. (1984) the stable inorganic P pool is initialized from the active P and is thought to be fourfold of the size of active P pool. However, the validity of this assumption for many soils has been recently challenged by Bolster and Vadas (2018). Research by Yuan et al. (2005) showed that the AnnAGNPS model failed to simulate dissolved P loss from a watershed in the USA, and concluded that this was likely due to simplistic assumptions made by Jones et al. (1984) regarding soil P processes. The original P routines of Jones et al. (1984) are based on inorganic P addition to soil over 6-month incubation studies (Sharpley et al. 1984, 1989), which cannot accurately simulate incidental P losses (water extractable P) and/or P dynamics associated with land application of manure/biosolids (Vadas et al. 2005; Collick et al. 2016). Collick et al. (2016) revised P routines of SWAT to consider P loss associated with application of manure to soil surface, but the revisions are not part of the publicly available version of SWAT and similar revisions for other process-based tools (e.g., AnnAGNPS) are still required.

An important parameter used in P routines of process-based tools to initialize soil P pools is P sorption capacity parameter (PSP) which represents the mass of P in soil solution once a relative equilibrium between solution P and active P pools is established. Sharpley et al. (1984, 1989) proposed equations to determine PSP based on physicochemical properties of soils that were subjected to three drying-wetting cycles over 6-month incubation studies. However, Vadas and White (2010) reported underestimation of soil total P pool by SWAT, partly due to overestimation of PSP values by equations proposed by Sharpley et al. (1984, 1989). Furthermore, unlike AnnAGNPS that calculates daily PSP values based on soil properties, SWAT and EPIC use a constant PSP value during the entire model simulation which may inaccurately predict soil P pools, particularly labile P pool (Vadas et al. 2013). Using accurate PSP of the soils is essential to predict the risk of P loss by these tools.

This highlights the crucial need for updating P routines of the process-based tools, and PSP values in particular, to accurately simulate soil P dynamics in agricultural watersheds. A significant research gap is a lack of effort to examine P routines of the Jones et al. (1984) and the underlying assumptions for cold climates. Importantly, in the method proposed by Sharpley et al. (1984, 1989) for determining PSP of USA soils, the soils were subjected only to three drying-wetting cycles during 6-month incubation studies. Consequently, the resulting PSP values could be invalid for soils in cold climates that undergo several freezing-thawing cycles, in addition to drying-rewetting cycles.

Hypothetically, soil P processes in cold climates are different from those processes in more temperate climates because in cold climates the soils often experience substantial abiotic perturbations. Frequent abiotic perturbations can potentially affect physical and biological properties of the soils, and consequently the mass of P loss in runoff (Blackwell et al. 2010; Vaz et al. 1994; Wall et al. 1988). During freezing, for example, water in soil pores requires more space as it expands to form ice crystals. This would apply pressure on soil pore walls resulting in disruption of soil aggregates, increased soil erodibility, and subsequent particulate P loss to runoff (Lehrsch et al. 1991; Wall et al. 1988). Freezing-thawing and/or drying-rewetting cycles can solubilize P from the soil microbial biomass through bacterial cell lysis, thereby increasing dissolved P loss to runoff (Blackwell et al. 2010). Furthermore, research has also shown that freezing-thawing cycles can significantly increase P release from plant residues depending on the freezing intensity, plant type, the moisture content of plant residue, the number of freeze-thaw cycles, and the level of interactions between soil and plant residue (Cober et al. 2018, 2019; Bechmann et al. 2005; Elliott 2013). Considering the effect of freezing-thawing cycles on partitioning between soil P pools and P movement through the landscape is of great importance.

### Empirical P loss assessment tools

Unlike process-based tools, empirical P loss assessment tools (i.e., P indices) do not attempt to model soil P dynamics to predict P loss in runoff. These tools are simplified to operate with a limited number of input parameters, so that they can practically be used by landowners or consultants rather than researchers and represent the annual risk of P loss rather than a calculated amount for each runoff event. Several P indices estimate the inherent risk of dissolved P losses from a field by multiplying agronomic soil P test value (e.g., Olsen extractable P concentration) by an extraction coefficient and runoff potential. Particulate P loss is also determined by multiplying the quantity of soil loss by mean P concentration in sediments (e.g., Reid 2011). Thus, the annual risk of P loss predicted by P indices largely depends on P concentration in soil/sediments.

The dynamic soil/sediment P status at CSAs is the balance of the amount of P applied to these areas, the mass of P removed by runoff and/or vegetation, and the mass of P deposited in these areas by runoff from upslope (Page et al. 2005). However, P indices ignore variations in soil P status with season and abiotic perturbations because they rely on a single P concentration value of soil/sediment. In cold climates for example, the potential of P loss to runoff from frozen/partially frozen soils during winter or early spring is assumed to be the same as P loss from soils to runoff in summer/fall, even though it may not be due to seasonal differences in soil microbial activity as well as the level of interactions between applied fertilizer/manure and soil. Since the time scale of P indices is annual, using a single P extraction coefficient may be adequate to predict the potential of P loss to runoff. However, if this coefficient is derived from growing season conditions and is actually quite different under winter conditions, the accuracy of this coefficient is called into question.

Phosphorus indices generally consider inherent and applied soil P sources separately to describe soil P dynamics and to identify areas with greater risk of P loss (e.g., Weld et al. 2002; Andersen and Kronvang 2006; Reid 2011). This separation approach to some extent overlooks the interaction between these sources and assumes applied P (manure and/or fertilizer) as a large P source with a short-term impact on water quality compared with inherent soil P which could be a small P source but with a continuous, long-term impact on downstream water quality (Kleinman et al. 2011a, b).

## Simulating runoff contributing areas to P loss by P loss assessment tools

Understanding hydrological processes governing runoff generation and movement within agricultural watersheds is critical for identifying CSAs. However, identifying the location of runoff generating areas in agricultural watersheds and their hydrological connections to surface waters is very challenging. Table 1 presents some of the P loss assessment tools and their approach for predicting runoff contributing areas and hydrological connectivity to surface waters. Since there is an overlap between some of the empirical and process-based P loss assessment tools in their approach for predicting runoff contributing areas, both of these tools were discussed in this section. The following sections describe various approaches used by current P loss assessment tools to predict runoff contributing areas of an agricultural watershed.

**Table 1:** Some of the P loss assessment tools and their approach for simulating runoff contributing areas to P loss in various climates

Tool category	Tool name	Spatial scale	Approach to predict runoff contributing areas	Approach to consider hydrological connectivity	Reference
Empirical tools	CADA-ECM	Watershed	Topographic index	Contribution of saturation-runoff areas to surface waters	Endreny and Wood 2003
	Danish P index	Watershed	Topsoil saturated hydraulic conductivity and slope steepness	Proximity to surface waters depending on the presence/absence of riparian buffer strip	Andersen and Kronvang 2006
	Modified Ontario P index	Field	Soil hydrological group and slope steepness	Proximity to surface waters	Reid 2011
	Norwegian P index	Field and sub-watershed	Soil hydraulic conductivity, slope steepness and the presence of tile drains	Proximity to surface waters depending on presence/absence of riparian buffer strip or grassed waterway	Bechmann et al. 2007
	Ohio P index	Field	Hydrologic soil group and slope steepness	Proximity to streams and/or the possibility of P transfer by concentrated flows to streams or tile inlet	Williams et al. 2017
	Pennsylvania P index	Field	Topsoil saturated hydraulic conductivity and slope steepness	Proximity to surface waters depending on the presence/absence of riparian buffer strip or grassed waterway	Sharpley et al. 2003b
	STEM-P	Watershed	Modified NRCS-CN equation	Calculating runoff travel times to surface waters by DHM-WM	Li et al. 2017
	Travel-time P index	Watershed	NRCS-CN equation	Calculating of runoff travel times to surface waters for overland flow, interflow and channel flow	Buchanan et al. 2013
Process-based tools	SWAT	Watershed	Modified NRCS-CN equation	Route runoff from HRU directly to the stream	Niraula et al. 2013
	GWLF	Watershed	NRCS-CN equation	Not stated	Niraula et al. 2013
	SWAT-VSA	Watershed	Reconceptualized NRCS-CN equation and topographic index	Route runoff from HRU directly to the stream	Collick et al. 2015
	AnnAGNPS	Watershed	NRCS-CN equation	Route runoff from a grid cell to another grid cell and finally to the stream	Li et al. 2015

*AnnAGNPS*, annualized agricultural non-point source pollution; *CADA-ECM*, Contributing areas and dispersal area export coefficient model; *DHM-WM*, distributed hydrological model for watershed management; *GWLF*, Generalized Watershed Loading Function; *NRCS-CN*, Natural Resources Conservation Service’s Curve Number.; *STEM-P*, spatially and temporally distributed empirical model for phosphorus management; *SWAT*, soil and water assessment tool; *SWAT-VSA*, soil and water assessment tool for variable source area; *HRU*, hydrological response unit

### Proximity to surface water approach

Many empirical P loss assessment tools rely on a predetermined distance from surface water as runoff contributing areas to P loss during rainfall or snowmelt events. This is associated with the general assumption and informal field observations that areas close to surface waters have a higher potential for P loss (e.g., Gburek et al. 2000; Sharpley et al. 2003b; Andersen and Kronvang 2006; Williams et al. 2017). For example, the Ontario P index assumes that runoff contributing areas of a field are limited to areas located within 50 and 150 m from surface water during growing and non-growing season, respectively (Reid 2011). This simplistic assumption regarding runoff contributing areas overlooks the effects of topography, event type (rainfall vs. snowmelt) and subsurface drainage on runoff generation, channelization, and hydrological connectivity of surface runoff (Thomas et al. 2016). For example, topography can aggregate diffuse shallow flows into concentrated flows leading to runoff with a high flow rate that can transfer P from a longer distance within a field from the surface water (Reid 2011; Habibiandehkordi et al. 2017). In cold climates, the majority of runoff and P loss generally occurs during spring-snowmelt events (e.g., Hansen et al. 2000; Glozier et al. 2006; Little et al. 2007) when the land is still frozen and soil infiltration is minimal. Our informal field observations in Manitoba and Ontario, Canada show that snowmelt runoff can be initiated from areas well beyond 150 m from surface waters. The proximity to surface water approach is also questionable where artificial drainage system or tile drains are present as these features can facilitate subsurface P transfer to water bodies from a greater distance from surface water (Reid et al. 2012).

### Topographic index approach

Past research has shown strong relationships between saturation-excess runoff contributing areas of a watershed and soil moisture patterns (e.g., Walter et al. 2000; Cheng et al. 2014). The topographic index (TI) proposed by Kirkby (1975) is widely used to predict such areas within a watershed with potential of saturation-excess runoff by taking into account the relationship between the upslope contributing area (α), and the local surface slope angle (β) as ln(α/tan β) (Beven 2001; Endreny and Wood 2003). Endreny and Wood (2003) used the topographic index (TI) to identify CSAs of P loss in the New York’s West Branch River watershed. However, the steady state assumption of TI does not address variations in the size of runoff contributing areas by season. The assumption also ignores the presence of artificial tile drains and/or bedrock topography that can alter saturation-excess runoff contributing areas (Beven 1997; Page et al. 2005; Freer et al. 2002).

Buchanan et al. (2013) proposed the travel-time P index (TTPI) that also relies on TI approach to determine saturation-excess runoff areas, but it considers hydrological connectivity based on the runoff travel time (Table 1). The travel-time between each saturation-excess runoff generating area and a receiving stream is determined from the depth of overland flow and the downslope flow conditions (Buchanan et al. 2013). However, both TI and TTPI values vary with digital elevation model (DEM) resolution (Quinn et al. 1991, 1995; Valeo and Moin 2000), and high-resolution DEM may not be available for many regions.

The TI approach also ignores the potential of P transfer from CSAs to surface waters through infiltration-excess runoff process, although this process can occur frequently in cold climates. In cold climates, soil infiltration is minimal when the ground is frozen in winter, thereby infiltration-excess runoff could occur even under medium intensity rainfall events. Despite recent efforts to optimize formulation of TI, for example by using a fine resolution (3 m) DEM and the multiple triangular slope algorithm to compute slope, the TI approach at its very best appeared to be capable of explaining only 60% of soil moisture patterns (i.e., saturation-excess runoff contributing areas) of agricultural landscapes in south-central New York, USA. where saturation-excess is the dominant runoff process (Buchanan et al. 2014).

### Curve Number and Green and Ampt approaches

Several process-based P loss assessment tools such as SWAT, the generalized watershed loading function (GWLF) model (Haith and Shoemaker 1987), and AnnAGNPS use the Natural Resources Conservation Service’s Curve Number (CN) (USDA-SCS 1972) or Green and Ampt (Green and Ampt 1911) equations to predict runoff contributing areas, assuming that runoff is generated only through infiltration-excess process (Table 1).

Consequently, these tools are unable to capture runoff contributing areas where saturation-excess is the dominant runoff generating process. For example, runoff generated from shallow permeable soils above a restricting layer and runoff over frozen/partially frozen soils are not well quantified using these models (Agnew et al. 2006; Easton et al. 2008). Effort has been made to reconceptualize the GWLF and SWAT models to account for the areas with potential of saturation-excess runoff (Easton et al. 2008; Schneiderman et al. 2007). For example, the modified version of SWAT known as the SWAT-VSA appears to be promising in representing the timing, quantity, and spatial distribution of surface runoff contributing areas compared with the traditional SWAT model (Collick et al. 2015; Easton et al. 2008), but its performance yet to be evaluated in cold climates.

## Considering hydrological connectivity by P loss assessment tools

Currently, neither empirical nor process-based P loss assessment tools can reliably consider hydrological connectivity between the landscape and surface water during runoff events. As mentioned earlier, empirical tools only consider areas close to streams/rivers as hydrologically connected areas to surface waters. The process-based tools assume that hydrologic response units (HRUs) or grid cells contribute equally to runoff generation and P loss and are not capable of simulating hydrological connectivity between discrete units of the landscape (Gassman et al. 2007). Generally, hydrological connectivity refers to the transfer of water from one part of the landscape to another that can lead to some watershed runoff response (Bracken and Croke 2007). This hydrological connectivity definition does not consider the physical and biogeochemical processes affecting the fate of P being transferred across hydrologically connected landscapes (Oldham et al. 2013). Therefore, we adopted and modified the "material connectivity" definition of Oldham et al. (2013) to a definition of material-P connectivity as the ability of P to transfer between landscape and receiving waters while subject to physical and biogeochemical processing. Although significant research has been undertaken to identify CSAs of P loss in agricultural watersheds over decades, our knowledge about material-P connectivity, particularly in cold climates is still in its infancy.

Understanding how material-P connectivity between landscape positions and surface waters develops and changes over time would ultimately lead to improving P loss assessment tools for accurately predicting CSAs in cold climates. In the Canadian prairies, for example, the presence of a large number of landscape depressions known as “prairie potholes” resulted from the glacial recession can significantly affect runoff generation and material-P connectivity (Casson et al. 2019; Hayashi et al. 2003; Wilson et al. 2019). Prairie potholes can intercept surface runoff, promote infiltration and deposition of particulate P, and provide an opportunity for biogeochemical removal from or release of P to surface runoff. During rainfall or snowmelt events, prairie potholes can be connected to drainage networks through fill-and-spill process (Leibowitz et al. 2016), and material-P connectivity between landscape and surface water may occur. The balance between the rate of P transport into the potholes, the rate of particulate P deposition, and the rate of P removal or release to surface runoff through biogeochemical processes (e.g., sorption to soil, reductive-dissolution of Al/Fe/Ca/Mg-bound P under reduced condition) will determine the material-P connectivity between the landscape and surface water. Mekonnen et al. (2016) developed a new version of SWAT, known as SWAT with probability distributed landscape depression (SWAT-PDLD), which is capable of simulating runoff in landscapes with numerous landscape depressions. However, neither the SWAT-PDLD nor other P loss assessment tools consider P retention or remobilization within landscape depressions.

An important step to develop a working model of material-P connectivity is explicitly identifying runoff contributing areas under seasonally varying weather conditions. Electrical resistance sensors appear to be a promising tool for recording the time of runoff initiation, identifying surface runoff contributing areas, and their connections to surface waters (Masselink et al. 2017; Moody and Martin 2015). High-resolution soil moisture measurements along with runoff and stream water chemistry analyses during rainfall and/or snowmelt events may reveal how hydrological connectivity affects nutrients export from land to water bodies (Ocampo et al. 2006; Stieglitz et al. 2003). Research has shown that the lack of hydrological connectivity between landscape components can lead to deposition of P-enriched sediments at specific locations within an agricultural field where they can be further transferred to surface waters by subsequent runoff events (Buda et al. 2009). Therefore, frequent and detailed soil sampling, as an indirect evidence, could reveal information for modeling purposes regarding material-P connectivity for the specific climate, soil, and topographical conditions.

## Summary and further research needs

Accurate identification of CSAs in agricultural watersheds using P loss assessment tools is needed to ensure cost-effective implementation of BMPs to reduce the risk of P transfer from land to water bodies. Current P management strategies in cold climates rely on the output of P loss assessment tools which may not fully represent hydrological and biogeochemical processes responsible for P loss in these regions. The concepts discussed in this paper should be used as a starting point for the modification and reconceptualization of P loss assessment tools for accurately identifying CSAs in cold climates.

Like many P indices, process-based P loss assessment tools also appear to have limited ability in accurately predicting P loss from the landscape, particularly in cold climates where runoff is mainly driven by snowmelt. This could be, in part, associated with the snowmelt hydrology component of these tools which cannot fully consider runoff over frozen land, intermittent snow melting process (i.e., when snow melts due to short-term warm temperature and refreezes before runoff reaches to surface water), and infiltration into partially frozen soils (see for example, Wang and Melesse 2005; Das et al. 2008; Lévesque et al. 2008). Improving the snowmelt hydrology component of process-based tools is necessary to accurately predict P loss from the landscape in cold climates.

There is lack of information in the literature on the ability of P routines of process-based tools to accurately initialize the quantity of soil P pools in cold climates; neither is there any attempt to update the equations used to estimate PSP values from soil properties in cold climates. In cold climates, the soils undergo several freezing-thawing and drying-rewetting cycles which could affect the quantity of soil P pools and subsequently the potential of P loss to runoff. Further research is needed to understand the quantity of soil P pools and the interactions between soil P biomass and P concentration in runoff for soils that experience substantial abiotic stresses.

There is a crucial need for developing P loss assessment tools capable of considering the magnitude, duration, and frequency of material-P connectivity between the landscape and surface waters under different conditions of topography, soil texture and chemistry, and management in cold climates. To develop such P loss assessment tools, techniques should be used to identify and quantify parts of the landscape that generate both surface and subsurface runoff under seasonally varying weather conditions. Furthermore, P loss assessment tools need to be improved to consider P retention or remobilizations as runoff moves through the landscape during rainfall and snowmelt runoff events.

Finally, while the process-based P loss assessment tools may better describe runoff and P loss from the landscape compared with empirical tools, they are complex and require a wide range of inputs that may not be readily available. Further, complex tools do not necessarily provide better information regarding runoff and P loss than simple P index approaches under diverse soil, climate and topography. Recent findings in the USA show that many P indices could provide information comparable with the complex, data-intensive process-based models (Osmond et al. 2017) which could be also the case in cold climates. This may be associated with the lack of frequent water quality data for model calibration. Such uncertainties associated with model parameters can result in equifinality, where combinations of input parameters by different models may lead to equally good fit to observations (Beven 2006). Regardless, evaluating P loss assessment tools with water quality monitoring data is crucial to assess whether changes in the structure and formulations of these tools result in improved delineation of CSAs in cold climates.

## Abbreviations

BMPs: Beneficial management practices
CSAs: Critical source areas
P: Phosphorus
